# Effects of urbanization-related environmental exposures on atopic dermatitis: A UK Biobank prospective cohort study stratified by genetic risk

**DOI:** 10.1016/j.jdin.2026.03.003

**Published:** 2026-03-18

**Authors:** Yangyiyi Yu, Ningling Wu, Chen Yang, Jinrong Zeng

**Affiliations:** a Department of Dermatology, Third Xiangya Hospital, Central South University, Changsha, Hunan, China; b Postdoctoral Station of Medical Aspects of Specific Environments, the Third Xiangya Hospital, Central South University, Changsha, China; c Department of Civil and Environmental Engineering, The Hong Kong Polytechnic University, Kowloon, Hong Kong, China; d Department of Chemical Materials, China CEC Engineering Corporation, Changsha, Hunan, China

**Keywords:** air pollution, atopic dermatitis, epidemiology, genetic susceptibility, green space, urban environmental exposures

## Abstract

**Background:**

Atopic dermatitis (AD) arises from complex gene-environmental interactions, and urban exposures are increasingly linked to its pathogenesis.

**Objective:**

To investigate the independent and interactive effects of urbanization-related exposures (air pollution, noise, traffic, green space) on AD incidence stratified by genetic susceptibility.

**Methods:**

Within the prospective UK Biobank cohort (*n* = 306,837), genetic risk was categorized by polygenic risk score. Associations and dose-response relationships were assessed using Cox and restricted cubic spline models.

**Results:**

Moderate and high air pollution mixtures were significantly associated with a higher AD risk, showing dose-dependent relationships. Higher green space coverage was associated with lower AD risk, particularly in the high genetic risk group (hazard ratio: 0.609, 95% confidence interval: 0.479-0.774). Noise was linked to an elevated AD risk exclusively in high genetic susceptibility, while urban residence and traffic load were associated with increased AD risk irrespective of genetic background. In a mutually adjusted multivariable model, the effects of traffic load and noise were attenuated.

**Limitations:**

Restricted ancestry, indoor pollutants, and residential mobility.

**Conclusion:**

Urban exposures show dose-dependent associations with AD risk. The assessment of urbanization exposure, in addition to genetic profiling, may represent a practical approach for population-level AD risk assessment and prevention.

**Capsule Summary**

• This study integrates findings by linking multiple urban environmental factors, beyond air pollution alone, to atopic dermatitis risk across genetic susceptibility groups.
• Because environmental risks persist regardless of genetic susceptibility, dermatologists should universally incorporate broad environmental assessments and protective lifestyle interventions into clinical practice.

## Introduction

Atopic dermatitis (AD) is a chronic inflammatory skin disorder affecting approximately 230 million individuals globally, with its age-standardized incidence rising annually.^1^^,^^2^ Prevalence exceeds 20% in children from industrialized nations (versus 10% in adults), significantly higher than rates in developing countries.^3^ Pronounced urban-rural disparities exist, with rural-raised children having substantially lower AD risks than urban counterparts,^4^ implicating urban environmental exposures as major pathogenic drivers. AD is clinically defined by intense pruritus, epidermal barrier dysfunction, eczematous lesions, and a vicious itch-scratch-barrier disruption cycle.^5^ High prevalence, chronicity, frequent comorbidities, and substantial patient burden necessitate prioritized prevention.

AD pathogenesis involves complex gene-environment interactions. While twin studies suggest heritability up to 75% and Genome-Wide Association Studies (GWAS) have identified susceptibility loci (eg, FLG, NLRP10),^6^, ^7^, ^8^, ^9^ the mechanisms by which environmental exposures modulate genetic susceptibility remain to be elucidated. This polygenic architecture defines the heritable component of AD pathogenesis and provides the molecular foundation for developing polygenic risk score (PRS)-based prevention strategies.

Beyond genetic susceptibility, urbanization-related factors are strongly implicated. The International Study of Asthma and Allergies in Childhood (ISAAC) revealed substantial geographic variation in AD prevalence among ethnically similar populations.^10^ Since the 1970s, urban areas have consistently demonstrated higher atopic disease incidence than rural regions. A recent global meta-analysis confirmed urban residence significantly increases AD risk compared to rural residence.^11^ Notably, such environmental drivers may extend beyond postnatal triggers to include prenatal insults.^12^

The pathogenesis of AD is complex and incompletely understood. External environmental factors promote AD development by compromising skin barrier function, inducing inflammatory responses, and amplifying pruritic signaling.^13^, ^14^, ^15^ Critically, interactions exist between environmental exposures and genetic susceptibility. Exposures like phthalates can interact with FLG variants to increase AD risk,^16^ highlighting differential responses to identical environmental stimuli across genetic backgrounds.

**Fig 1:**
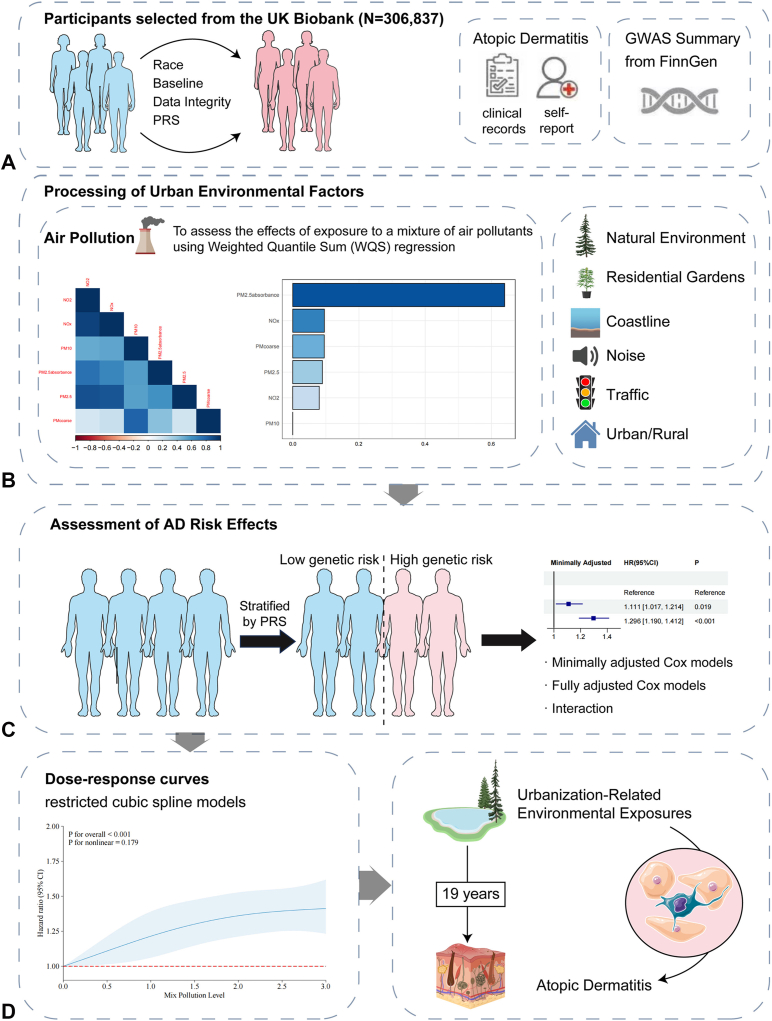
Overall study design. **A,** Participants were selected from the UK Biobank (*N* = 306,837). **B,** Processing of urban environmental factors involves assessment of mixed air pollutant exposure using weighted quantile sum (WQS) regression, and inclusion of natural environment, residential gardens, coastline, noise, traffic, and urban/rural status. **C,** Assessment of AD risk effects, stratified by genetic risk. Analyses include minimally adjusted Cox models, fully adjusted Cox models, and interaction analyses. **D,** Dose-response curves of urbanization-related environmental exposures derived from restricted cubic spline models.

Although research in recent years has explored the contributions of environmental exposures and genetic susceptibility to various diseases,^17^^,^^18^ including the onset of AD,^19^ significant knowledge gaps remain. Studies have not mapped the effects of multimodal urban exposures alongside genetic susceptibility. To address this, we integrated urbanization-related environmental metrics with PRS, including air pollution, green space, noise, and traffic load. We aimed to systematically disentangle the independent and interactive effects of these exposures on AD development, providing a theoretical foundation for precision prevention. The overall study design is shown in Fig 1.

## Methods

### Study population

Data were obtained from the UK Biobank prospective cohort (Application 679050).^20^ Between 2006 and 2010, over 500,000 participants aged 40 to 70 were recruited across 22 UK centers. Ethical approval was granted by the North West Multi-Centre Research Ethics Committee.

### AD diagnosis

Incident AD cases were identified via ICD-10 coding (L20) from hospital admission and primary care records, supplemented by self-reports of physician-diagnosed AD.^21^ Participants with prevalent AD at baseline were excluded. Follow-up extended from baseline to diagnosis, death, or censoring (January 1, 2024).

### Environmental exposures

Air pollutant concentrations (μg/m^3^) were estimated using standardized Land Use Regression (LUR) models (ESCAPE project) developed for the UK Biobank cohort.^22^, ^23^, ^24^ A weighted quantile sum (WQS) regression index integrated the 6 pollutants into a single weighted metric.^25^

“Natural environment” (proportion of non-built-up area) and “Greenspace” (officially classified green land) were calculated within 300 m and 1000 m residential buffers using GLUD and CEH data, consistent with previous studies.^26^, ^27^, ^28^ Distance to the coastline (km) was used directly as a continuous measure.

The 24-hour average sound pressure levels were modeled using the Common Noise Assessment Methods (CNOSSOS-EU)^29^ and categorized as low (30-59 dB), medium (60-89 dB), or high (>90 dB). Traffic variables were computed within a Geographic Information System during the LUR process. Traffic load was assessed via proximity to major roads (binary) and distance tertiles. Urban/rural residence was defined by the Office for National Statistics.

### PRS calculation

To quantify genetic susceptibility, a PRS was constructed using GWAS summary statistics from the FinnGen consortium (Release 12). PRS have proven effective for assessing the cumulative effects of various risk-associated variants and are used to quantify genetic susceptibility to polygenic disorders.^30^ The validity and predictive power of a PRS are highly dependent on the ancestral alignment between the discovery cohort and the target cohort. This is due to population-specific differences in allele frequencies, linkage disequilibrium patterns, and potential gene-environment or gene-gene interactions.^31^ To minimize population stratification bias and ensure ancestral alignment, the target sample was restricted to White British/Irish UK Biobank participants. This group provided the closest feasible ancestral match to the European-based FinnGen discovery sample. This necessary methodological constraint is critical for ensuring the reliability of genetic risk stratification. We identified 42 independent genome-wide significant single-nucleotide polymorphisms (SNPs) (*P* < 5 × 10^−8^; Supplementary Table I, available via Mendeley at https://data.mendeley.com/datasets/v5gsrvvc8z/1). PRS for each participant was calculated by summing the allele counts weighted by effect sizes (beta coefficients), and participants were subsequently stratified into high and low genetic risk groups based on the median PRS.

### Covariates

Covariates were selected based on prior knowledge, as previous studies have demonstrated that these factors are potential risk factors for AD, serving as potential confounders.^2^^,^^32^, ^33^, ^34^, ^35^, ^36^, ^37^, ^38^ These included age, sex, body mass index (BMI), smoking status (current/former/never), alcohol intake frequency, weekly physical activity, educational attainment, annual household income, and ethnicity.

### Statistical analysis

Continuous variables are expressed as mean ± standard deviation (SD). Categorical variables are presented as number (%). Baseline differences were assessed using analysis of variance (ANOVA) or the Chi-square test, as appropriate. The absolute incidence of AD was calculated as the number and percentage within each categorical group. Associations between exposures and incident AD were evaluated using multivariable Cox proportional hazards models, reporting hazard ratios (HRs) with 95% confidence intervals (CIs). Two models were constructed: a minimally adjusted model (age, sex, BMI) and a fully adjusted model (age, sex, BMI, smoking status, educational attainment, mean annual total household income before tax, and ethnicity). To address correlation among exposures, a comprehensive model including all significant environmental factors was constructed, with multicollinearity assessed via Variance Inflation Factor (VIF). Furthermore, we ranked the variables based on their Wald Chi-square statistics derived from this model. Exposure-response relationships were examined using restricted cubic splines (RCS). Additionally, the association between PRS and AD risk was also evaluated using RCS to verify the linearity assumption. Gene-environment interactions was tested by including an interaction term (PRS × exposure) in the Cox models.^39^ Sensitivity analyses employed an enhanced adjustment model (age, sex, BMI, smoking status, and mean annual total household income before tax). Statistical analyses were performed using R (version 4.2.1), with a 2-sided *P*-value < .05 considered significant.

## Results

### Study population

From 502,467 participants, the final cohort comprised 306,837 individuals after exclusions: (1) 30,095 with incomplete data or non-White British ancestry; (2) 160,313 with prevalent AD at baseline; and (3) 5222 with genetic data mismatches (Supplementary Fig 1, available via Mendeley at https://data.mendeley.com/datasets/v5gsrvvc8z/1). During a median follow-up of 19.0 years, 3173 incident AD cases were identified. Baseline characteristics differed significantly between cases and non-cases regarding smoking, sex, age, income, education, and ethnicity (Table I). The PRS showed a linear dose-response relationship with AD risk (*P* for nonlinearity = .079, Supplementary Fig 2, available via Mendeley at https://data.mendeley.com/datasets/v5gsrvvc8z/1), supporting the use of standard interaction terms.

**Table I:** Comparison of demographic and socioeconomic characteristics between atopic dermatitis cases and non-cases

Variables	Total (*n* = 306,387)	Atopic dermatitis (*n* = 3173)	No atopic dermatitis (*n* = 303,214)	*P*
Smoking status, *n* (%)				<.001
Never	177,826 (58.04)	1716 (54.08)	176,110 (58.08)	
Previous	118,737 (38.75)	1357 (42.77)	117,380 (38.71)	
Current	8635 (2.82)	87 (2.74)	8548 (2.82)	
Unknown	1189 (0.39)	13 (0.41)	1176 (0.39)	
Number of days week of moderate physical activity, *n* (%)				.468
High (5,6,7)	116,035 (37.87)	1165 (36.72)	114,870 (37.88)	
Low (0,1)	58,817 (19.2)	626 (19.73)	58,191 (19.19)	
Moderate (2,3,4)	117,367 (38.31)	1224 (38.58)	116,143 (38.3)	
Unknown	14,168 (4.62)	158 (4.98)	14,010 (4.62)	
Number of days week of vigorous physical activity, *n* (%)				.395
High (5,6,7)	35,946 (11.73)	363 (11.44)	35,583 (11.74)	
Low (0,1)	148,116 (48.34)	1528 (48.16)	146,588 (48.34)	
Moderate (2,3,4)	108,569 (35.44)	1120 (35.3)	107,449 (35.44)	
Unknown	13,756 (4.49)	162 (5.11)	13,594 (4.48)	
Sex, *n* (%)				<.001
Female	168,071 (54.86)	1848 (58.24)	166,223 (54.82)	
Male	138,316 (45.14)	1325 (41.76)	136,991 (45.18)	
Age, mean ± SD	57 ± 7.99	57.59 ± 7.89	56.99 ± 7.99	<.001
Average total household income before tax, *n* (%)				<.001
Less than 18,000	56,361 (18.4)	655 (20.64)	55,706 (18.37)	
18,000-30,999	68,062 (22.21)	694 (21.87)	67,368 (22.22)	
31,000-51,999	69,800 (22.78)	644 (20.3)	69,156 (22.81)	
52,000-100,000	54,742 (17.87)	521 (16.42)	54,221 (17.88)	
Greater than 100,000	14,536 (4.74)	151 (4.76)	14,385 (4.74)	
Unknown	42,886 (14)	508 (16.01)	42,378 (13.98)	
BMI, mean ± SD	27.41 ± 4.74	27.48 ± 4.78	27.41 ± 4.74	.429
Alcohol drinker status, *n* (%)				.442
Unknown	241 (0.08)	1 (0.03)	240 (0.08)	
Never	9806 (3.2)	94 (2.96)	9712 (3.2)	
Previous	9759 (3.19)	114 (3.59)	9645 (3.18)	
Current	286,581 (93.54)	2964 (93.41)	283,617 (93.54)	
Education level, *n* (%)				.022
College or university degree	32,265 (10.53)	362 (11.41)	31,903 (10.52)	
A levels/AS levels or equivalent	8221 (2.68)	83 (2.62)	8138 (2.68)	
O levels/GCSEs or equivalent	57,881 (18.89)	607 (19.13)	57,274 (18.89)	
CSEs or equivalent	21,597 (7.05)	183 (5.77)	21,414 (7.06)	
NVQ or HND or HNC or equivalent	43,301 (14.13)	422 (13.3)	42,879 (14.14)	
Other professional qualifications eg: nursing, teaching	89,351 (29.16)	917 (28.9)	88,434 (29.17)	
Unknown	53,771 (17.55)	599 (18.88)	53,172 (17.54)	
Ethnicity, *n* (%)				.008
White	283 (0.09)	3 (0.09)	280 (0.09)	
British	288,321 (94.1)	2944 (92.78)	285,377 (94.12)	
Irish	7537 (2.46)	105 (3.31)	7432 (2.45)	
Any other white background	10,246 (3.34)	121 (3.81)	10,125 (3.34)	

*SD*, Standard deviation.

### Air pollutant mixture

**Fig 2:**
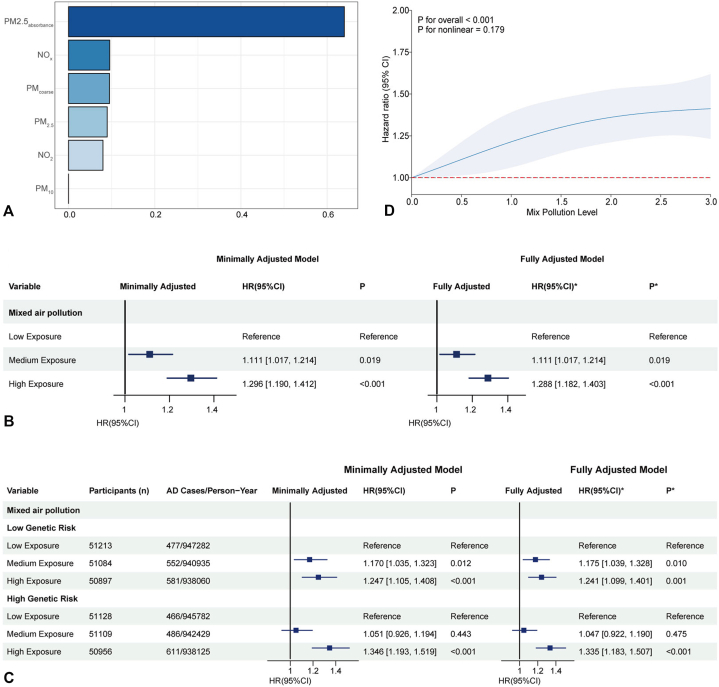
Associations between mixed air pollution exposure and AD risk. **A,** Weight contributions of air pollutants to AD risk from weighted quantile sum (WQS) regression model. The plot displays the relative weights of 6 air pollutants. **B,** Hazard ratios (HRs) and 95% confidence intervals (CIs) for AD risk across tertiles of mixed air pollution exposure in minimally and fully adjusted models. **C,** Stratified analysis by genetic risk: HRs and 95% CIs for AD risk across mixed air pollution exposure tertiles in low and high genetic susceptibility groups, in minimally and fully adjusted models. **D,** Restricted cubic spline (RCS) model illustrating the dose-dependent relationship between mixed air pollution mixture index and AD risk, with *P* values for overall and nonlinear associations. Minimally Adjusted Model: Adjusted by sex, age and BMI. ∗Fully Adjusted Model: Adjusted by sex, age and BMI, and further adjusted by smoking status, Average total household income before tax, Education level and Ethnicity. *CI*, Confidence interval; *HR*, hazard ratio.

Assessment of air pollutants revealed significant positive correlations among the 6 pollutants: NO_2_, NO_x_, PM_10_, PM_2.5_ absorbance, PM_2.5_, and PM_coarse_ (Supplementary Fig 3, available via Mendeley at https://data.mendeley.com/datasets/v5gsrvvc8z/1). The WQS model identified PM_2.5_ absorbance, NO_x_, and PM_coarse_ as the largest contributors to the air pollution mixture (Fig 2, *A*). In fully adjusted models, medium (HR = 1.111, 95% CI: 1.017-1.214) and high (HR = 1.288, 1.182-1.403) air pollution mixtures were associated with increased AD risk compared to the low tertile (Fig 2, *B*). The risk increased with higher exposure across both low and high genetic risk strata. In the low genetic risk group, medium and high exposures yielded HRs of 1.175 (1.039-1.328) and 1.241 (1.099-1.401); in the high genetic risk group, high exposure yielded an HR of 1.335 (1.183-1.507) (Fig 2, *C*). The interaction between the air pollution mixture and PRS was not significant (Supplementary Table II, available via Mendeley at https://data.mendeley.com/datasets/v5gsrvvc8z/1). RCS modeling indicated an approximately linear dose-response relationship (*P* < .001) (Fig 2, *D*).

### Green environment

**Fig 3:**
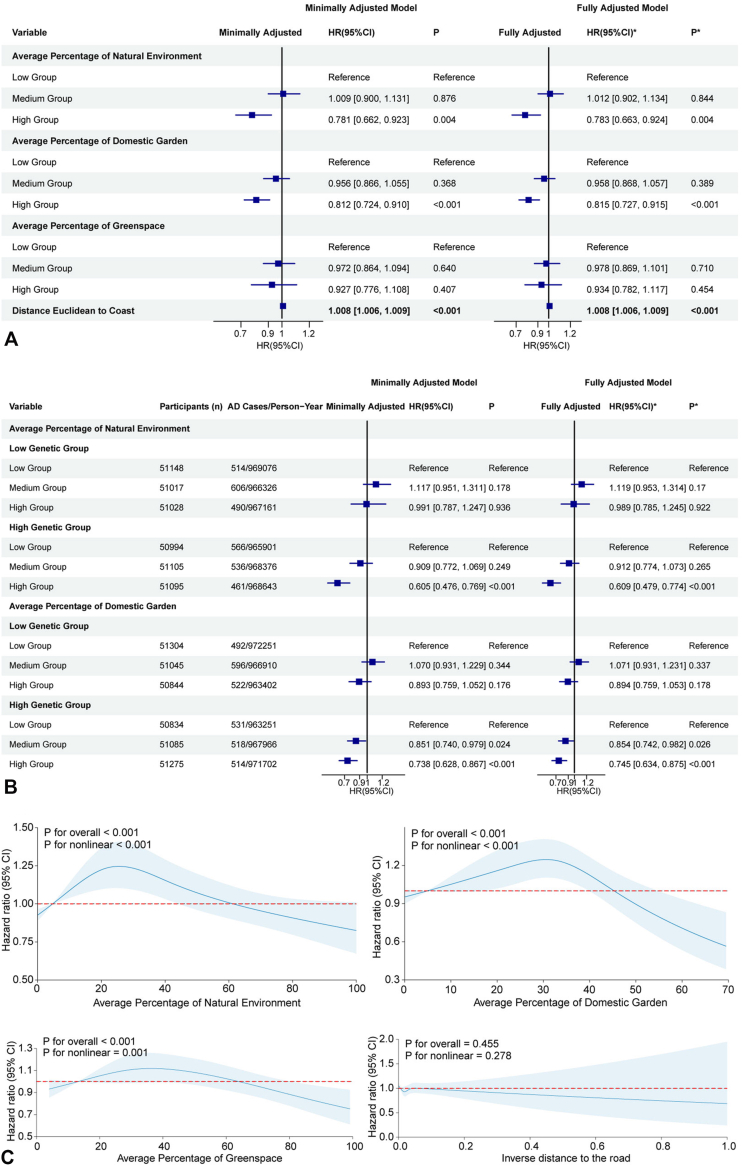
Associations between green environment exposure and AD risk. **A,** Hazard ratios (HRs) with 95% confidence intervals (CIs) for AD risk across tertiles of natural environment, domestic garden, greenspace, and distance Euclidean to coast in minimally and fully adjusted models. **B,** PRS-stratified analysis: HRs (95% CIs) for AD risk across green environment exposure levels in low and high genetic susceptibility groups, in minimally and fully adjusted models. **C,** Restricted cubic spline (RCS) models showing dose-dependent relationships between natural environment area, domestic garden proportion, greenspace coverage, inverse distance to the road and AD risk, with *P* values for overall and nonlinear associations. Minimally Adjusted Model: Adjusted by sex, age and BMI. ∗Fully Adjusted Model: Adjusted by sex, age and BMI, and further adjusted by smoking status, Average total household income before tax, Education level and Ethnicity. *AD*, Atopic dermatitis; *CI*, confidence interval; *HR*, hazard ratio.

High natural environment coverage and high domestic garden proportion were protective (HR = 0.783, 95% CI: 0.663-0.924 and HR = 0.815, 95% CI: 0.727-0.915, respectively), while general greenspace coverage showed no association (Fig 3, *A*). Shorter distance to the coast was associated with increased risk (HR per km decrease = 1.008, 95% CI: 1.006-1.009, *P* < .001) (Fig 3, *A*). In PRS-stratified analysis, the protective effects of high natural environment and domestic garden coverage were significant only in the high genetic risk group (HR = 0.609, 95% CI: 0.479-0.774; and HRs of 0.854 [0.742-0.982] for medium and 0.745 [0.634-0.875] for high garden coverage, respectively) (Fig 3, *B*), though formal interaction tests were non-significant (Supplementary Table II, available via Mendeley at https://data.mendeley.com/datasets/v5gsrvvc8z/1). Effects of greenspace and distance to coast were not modified by PRS (Supplementary Fig 4, available via Mendeley at https://data.mendeley.com/datasets/v5gsrvvc8z/1). RCS curves revealed nonlinear dose-response relationships for green exposures (Fig 3, *C*).

### Noise, urbanicity, and traffic

**Fig 4:**
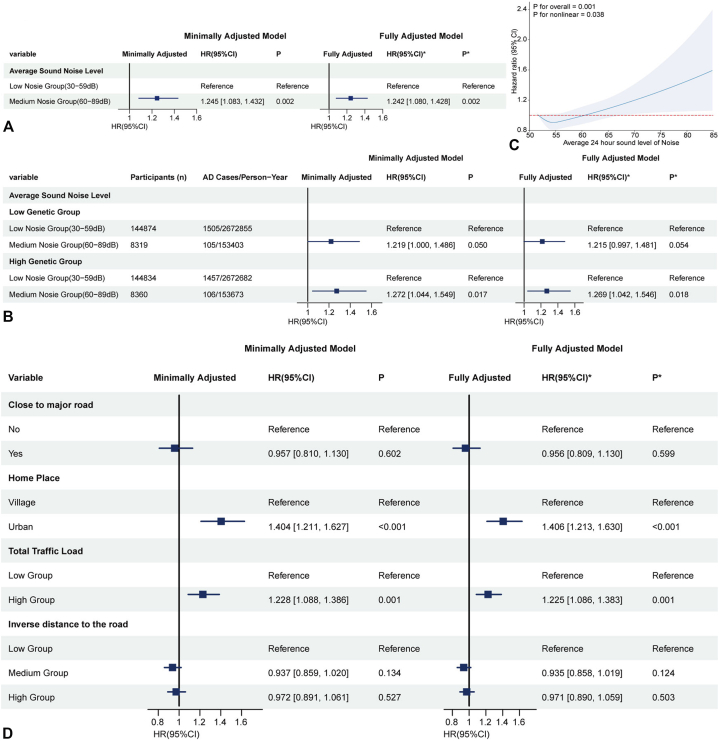
Associations between environmental noise exposure and AD risk. **A,** Hazard ratios (HRs) and 95% confidence intervals (CIs) for AD risk across noise exposure levels in minimally and fully adjusted models. **B,** Stratified analysis by genetic risk: HRs and 95% CIs for AD risk with moderate noise exposure in low and high genetic susceptibility groups, in minimally and fully adjusted models. **C,** Restricted cubic spline (RCS) model showing the dose-response relationship between average 24-hour sound noise level and AD risk, with *P* values for overall and nonlinear associations. **D,** HRs and 95% CIs for AD risk in relation to residential proximity to major roads, urban vs rural residence, traffic load (low vs high groups), and inverse distance to roads (low, medium, and high groups), presented in minimally and fully adjusted models. Minimally Adjusted Model: Adjusted by sex, age and BMI. ∗Fully Adjusted Model: Adjusted by sex, age and BMI, and further adjusted by smoking status, Average total household income before tax, Education level and Ethnicity. *CI*, Confidence interval; *HR*, hazard ratio.

Moderate noise exposure (60-89 dB) was associated with increased AD risk (HR = 1.242, 95% CI: 1.080-1.428) (Fig 4, *A*), particularly in the high genetic risk group (HR = 1.269, 95% CI: 1.042-1.546) (Fig 4, *B*); high exposure (≥90 dB) could not be reliably estimated due to limited power. RCS indicated a positive, quasi-linear dose-response above ∼60 dB (Fig 4, *C*). The interaction was not significant (Supplementary Table II, available via Mendeley at https://data.mendeley.com/datasets/v5gsrvvc8z/1).

Urban residence (vs rural) (HR = 1.406, 1.213-1.630) and high traffic load (HR = 1.225, 1.086-1.383) were associated with increased AD risk (Fig 4, *D*) irrespective of genetic background (Supplementary Fig 5, available via Mendeley at https://data.mendeley.com/datasets/v5gsrvvc8z/1). Inverse distance to the nearest major road was not associated with risk (Fig 4, *D* and Supplementary Fig 6, available via Mendeley at https://data.mendeley.com/datasets/v5gsrvvc8z/1). No significant interactions were found (Supplementary Table II, available via Mendeley at https://data.mendeley.com/datasets/v5gsrvvc8z/1). Sensitivity analyses confirmed the robustness of the primary findings (Supplementary Tables III-VI, available via Mendeley at https://data.mendeley.com/datasets/v5gsrvvc8z/1).

### Independent contributions

**Fig 5:**
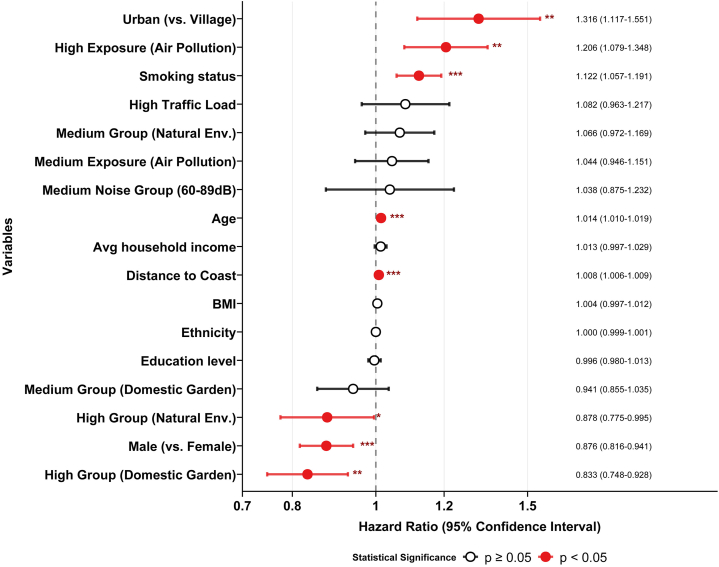
Independent effects of concurrent urban environmental exposures on AD risk. The multivariable Cox regression model simultaneously adjusting for all significantly positive environmental factors and covariates. Hazard ratios (HRs) and 95% confidence intervals (CIs) indicate the independent effect of each exposure on AD risk. ∗*P* < .05, ∗∗*P* < .01, ∗∗∗*P* < .001.

In a mutually adjusted multivariable model (Fig 5), urban residence (HR = 1.316, 95% CI: 1.117-1.551), high air pollution mixture (HR = 1.206, 95% CI: 1.079-1.348), and distance to coast (HR = 1.008, 95% CI: 1.006-1.009) remained significant risk factors. Conversely, high natural environment coverage (HR = 0.878, 95% CI: 0.775-0.995) and domestic garden proportion (HR = 0.833, 95% CI: 0.748-0.928) retained independent protective effects. Notably, the effects of traffic load and noise were attenuated in the multivariable model (Fig 5), suggesting their influence might be partially mediated by or shared with other urban factors.

Distance to the coast, age, and air pollution were identified as the strongest predictors in the multivariable model (Supplementary Fig 7, available via Mendeley at https://data.mendeley.com/datasets/v5gsrvvc8z/1). The VIF analysis showed low collinearity among variables (Supplementary Table VII, available via Mendeley at https://data.mendeley.com/datasets/v5gsrvvc8z/1), indicating that these environmental variables are statistically distinct.

## Discussion

This large prospective cohort study systematically evaluated the associations between urbanization-related environmental exposures and the risk of incident AD. Our central finding is that beyond the widely studied air pollution, other urban factors—noise, residential type, traffic load, and green space—were independently associated with AD incidence. Overall, composite air pollution exposure, noise, urban residential, and traffic load significantly increased AD risk, whereas natural environment coverage and domestic gardens were protective. Most associations exhibited threshold effects.

While the link between urbanization and respiratory allergy is well-established, evidence for AD has largely focused on air pollution.^40^ Evidence for other common urban exposures is sparse. To our knowledge, no large-scale prospective studies have specifically examined the associations of green environment exposure, urban-rural residence, and traffic load with AD incidence. Although observational studies suggest biodiverse microbial exposures and residential greenery may protect against allergies and asthma,^41^^,^^42^ and isolated reports like the National Health Insurance Service study link nighttime noise exposure to AD hospitalization duration,^43^ a fragmented, single-exposure approach dominates the field. Our findings provide the first comprehensive evidence for their independent roles in AD, advancing the field beyond a narrow focus on air quality towards an integrative “urban exposure” perspective.

While point estimates for environmental risks (eg, noise, lack of green space) were often higher in the high genetic risk group, interaction tests were non-significant, consistent with a recent study on PM_2.5_ and elderly-onset AD.^30^ This suggests that while genetic background influences baseline susceptibility, these environmental exposures exert a robust, direct influence on AD risk across the genetic spectrum. Conversely, risks for urban residence and high traffic load were consistent across genetic strata, highlighting their role as pervasive environmental risk factors. This aligns with recent critiques that GWAS-based risk stratification has limited explanatory power in the presence of strong environmental drivers.^31^^,^^44^

Pathogenic effects are likely mediated through pathways not captured by PRS, such as epigenetic modification, direct barrier disruption, and alterations to the skin and gut microbiome.^13^^,^^14^^,^^45^ Supporting evidence indicates pollutants can disrupt structural proteins, induce oxidative stress, and alter commensal microbes.^46^, ^47^, ^48^ Crucially, environmental toxicants can be ingested or translocated to the gut, inducing dysbiosis and reducing anti-inflammatory short-chain fatty acids.^49^^,^^50^ This disruption of the “gut-skin axis” promotes systemic type-2 inflammation, which compromises the epidermal barrier and exacerbates AD pathogenesis.^51^ Furthermore, environmental exposures may exacerbate underlying defects in epidermal lipid metabolism, a hallmark of AD.^52^

Regarding the strength of associations, while the large sample size of the UK Biobank enables the detection of modest effects, our primary exposures, such as urban residence (HR ∼1.41) and high air pollution (HR ∼1.29), demonstrated substantial effect sizes that are clinically relevant in environmental epidemiology. For continuous variables like distance to the coast (HR = 1.008), the seemingly small effect estimate reflects the risk change per single kilometer; the cumulative risk across the full exposure range would be considerable. Furthermore, consistent with Rose’s paradigm,^53^ widespread environmental exposures with low-to-moderate relative risks can generate a significant population-level disease burden due to the ubiquity of exposure.

Strengths of this study include the prospective design, large sample size, and precise exposure-outcome temporality, minimizing reverse causation. We provide novel evidence for dose-response relationships between multiple urban factors and AD. Limitations include the restriction to White British ancestry (to ensure PRS validity), which limits generalizability to other ethnic groups. While this approach maximizes the accuracy of genetic risk prediction within our study, it limits the generalizability of our findings to other populations globally. It is well-documented that genetic risk factors and disease prevalence can vary across different ancestries. Applying our current model to other ethnic groups would be inappropriate. Additionally, the lack of indoor pollutant data and the inability to account for residential mobility during follow-up are constraints. Although we could not account for residential mobility or duration of residence during follow-up, previous work in UK Biobank has shown that this cohort is residentially very stable, with over 70% of residential addresses remaining unchanged during the study's follow-up period.^54^, ^55^, ^56^ Future studies should incorporate epigenomics to elucidate mechanism-specific methylation patterns and validate these findings in diverse populations.

## Conclusion

This study demonstrates dose-dependent associations between multiple urbanization-related exposures and AD incidence. Air pollution, noise, urban residence, and traffic load increase risk, while green space confers protection. These risks persist across genetic susceptibility profiles, underscoring the primacy of environmental factors in AD etiology. Integrating urban exposure profiles with genetic risk may represent a practical approach for population-level AD prevention.

### Declaration of generative AI and AI-assisted technologies in the writing process

During the preparation of this work the authors used DeepSeek-V3.2 in order to improve readability and language. After using this tool, the authors reviewed and edited the content as needed and take full responsibility for the content of the published article.

## Conflicts of interest

None disclosed.
